# Unveiling the dark side of eating disorders: evidence on the role of dark triad and body uneasiness in youth

**DOI:** 10.3389/fpsyg.2024.1437510

**Published:** 2024-12-16

**Authors:** Marco Giancola, Simonetta D’Amico, Maria Giulia Vinciguerra

**Affiliations:** Department of Biotechnological and Applied Clinical Sciences, University of L’Aquila, L’Aquila, Italy

**Keywords:** dark triad, eating habits, body image, personality, health, mediation

## Abstract

Eating disorders comprise an array of mental disturbance with profound implications for individuals’ psychophysical and societal well-being. Extensive research has elucidated the role of the Big Five personality traits in explaining individual differences in the risk of eating disorders, overshadowing alternative personality taxonomies, such as the Dark Triad - DT (i.e., Machiavellianism, psychopathy, and narcissism). Accordingly, the current study aimed to address the association between DT and the risk of eating disorders, also exploring the potential involvement of body uneasiness as captured in terms of weight phobia (fear of being or becoming fat), body image concerns (worries related to physical appearance), avoidance (body image related avoidance behaviors), compulsive self-monitoring (compulsive checking of physical appearance), and depersonalization (detachment and estrangement feelings towards one’s own body). By using an online cross-sectional design, data were gathered from 419 participants. Results indicated that among the three dimensions of DT, only narcissism exhibited a positive correlation with the risk of eating disorders, while only weight phobia mediated this association. These findings yield theoretical implications extending the knowledge about the role of DT and body uneasiness in the risk of eating disorders. These results also have implications for tailoring prevention or treatment strategies to reduce the risk of eating disorders. Limitations and avenues for future research directions are discussed.

## 1 Introduction

Eating disorders encompass a broad array of severe and complex mental disturbances (e.g., anorexia nervosa, bulimia nervosa, binge-eating disorder), which profoundly impact individuals’ physical, psychological, and societal well-being (American Psychiatric Association, 2022). These disorders can affect people of all ages (although a high prevalence persists in adolescents and young adults), ethnicities, genders, and sexes (Halbeisen et al., 2022) and include a specific pattern of characteristics, such as repetitive eating behaviors, food restrictions, and dysfunctional body image perceptions (Siddiqi et al., 2024). Eating disorders can determine a wide variety of health problems, including obesity, type-2 diabetes, hypertension, high cholesterol, heart disease, gallbladder disease, or even death (Bourdier et al., 2018). Indeed, they have the second highest mortality rate of any psychiatric disease, second only to the opioid crisis (Arcelus et al., 2011; Iwajomo et al., 2021). Therefore, as their nature, eating disorders can be challenging to manage due to the high relapse cases and chronic nature (Keel and Brown, 2010). Research indicated that around 1.9% of people in Western countries suffer from eating disorders, and the rate is higher among women, at up to 2.6% (Mayer et al., 2024). Similarly, studies from Asian countries, such as mainland China, Singapore, and Iran, revealed that the prevalence of eating disorders appears to be comparable to–or perhaps even higher than – that observed in Western Countries (Kim et al., 2021). Other studies indicated that over the period between 2000 and 2018, the point prevalence of eating disorders increased from 3.5% for 2000–2006 to 4.9% for 2007–2012 and 7.8 for the 2013–2018 period (Galmiche et al., 2019). Due to the enhancement of the prevalence as well as their persistence and dangerous nature, eating disorders pose significant challenges for healthcare systems as well as society as a whole (Johns et al., 2019).

One of the most pressing issues in addressing eating disorders is the tendency for affected individuals to deny or conceal their illness and avoid professional assistance (Smink et al., 2012). This reluctance to acknowledge the problem and pursue help not only delays effective treatment but also exacerbates the severity and chronicity of the disorders (Gioia et al., 2024). Consequently, there is an urgent need to proactively address eating disturbances by understanding the multifaceted mechanisms underlying their aetiology, symptomatic expression, and maintenance.

In the field of psychology, research devoted much attention on the main predictors of eating disorders, stressing the critical role of personality (Meneguzzo et al., 2021). For instance, some studies indicated a critical role of borderline, perfectionistic, and paranoid personality traits in the development of clinical profiles that are closely related to eating disorders, including (Khosravi, 2020; Lasson and Raynal, 2021; Longo et al., 2024). In addition, a wide array of studies focused on the association between eating disorders and the Big Five personality traits, namely Openness, Conscientiousness, Extraversion, Agreeableness, and Neuroticism. Specifically, research has consistently indicated the critical role of high levels of Neuroticism and low degrees of Extraversion in explaining the individual disposition toward the risk of eating disorders (Clarke and Kiropoulos, 2021; Farstad et al., 2016; Ghaderi and Scott, 2000; Miller et al., 2006). Nonetheless, this focus on the Big Five overshadowed other personality taxonomies, such as the Dark Triad (DT), which may also contribute to explaining individual differences in eating habits and eating disorder symptomatology (Giancola et al., 2024a).

DT comprises Machiavellianism, psychopathy, and narcissism, which represent three distinct yet interrelated subclinical maladaptive and socially aversive personality traits (Paulhus and Williams, 2002; Giancola et al., 2023a). People who scored highly in Machiavellianism exhibit self-centeredness, cynicism, exploitativeness, and manipulativeness (Došenović and Dinić, 2024; Jones, 2016), while those high in psychopathy demonstrate callousness, thrill-seeking behaviors, interpersonal antagonism as well as a lack of empathy, remorse, and anxiety (Patrick et al., 2012). Narcissism, instead, is characterized by high self-absorption, dominance, a sense of entitlement, and grandiosity (Back et al., 2013). Although research on DT has traditionally focused on its implication for morality, antisocial behaviors and interpersonal relationships, such as romantic relationships and friendships (Brewer et al., 2023; Giancola et al., 2024b; Sijtsema et al., 2019), emerging studies also suggested the relevance of DT to eating behaviors and disorders.

Research on eating habits suggested that individuals with high DT exhibit a pronounced inclination toward distinct patterns of eating habits and attitudes. For instance, some studies suggested that people high in DT tend to embrace an omnivore eating style or meat consumption and meat-eating justification strategies rather than vegan or vegetarian choices (Mertens et al., 2020; Mertens et al., 2021; Sariyska et al., 2019).

Of relevance for eating disorders, Brunett and Oberle (2022) found that individuals with high levels of DT tend to have a greater pathological fixation on consuming only biologically pure and healthy food (i.e., orthorexia nervosa). In addition, psychopathy was found to be associated with uncontrolled eating in both sexes and a higher disposition to overeat in response to negative emotions due to an inability to resist emotional cues (i.e., emotional eating) in men (Shi et al., 2022). Similarly, narcissism was found to be positively associated with both eating disorder symptoms, a desire to have a muscular body (Szymczak et al., 2023) and higher uncontrolled eating in men (Shi et al., 2022). This means that people with high narcissism are more likely to be focused on their physical appearance and striving to achieve an ideal body (Szymczak et al., 2023). As concerns Machiavellianism, although a recent study (Shi et al., 2022) indicated that this trait may represent a protective factor against the inclination to overeat when women experience a loss of control over food intake, other studies consistently showed positive associations between Machiavellianism and some facets of pathological eating, such as orthorexia nervosa (Brunett and Oberle, 2022) and anorexia nervosa (Hambrook and Tchanturia, 2008). For instance, Hambrook and Tchanturia (2008) argued that Machiavellianism and anorexia nervosa share deception and manipulation that is advantageous for an individual with eating disorders to evade detection and suspicion regarding their condition.

Despite this evidence, the findings regarding the role of DT in the risk of dysfunctional eating remain limited, while the mechanisms potentially involved in this association have been largely unexplored until now. This gap in knowledge underscores the importance of further research to elucidate the complexity of the association between personality and eating behaviors.

In this context, body uneasiness could emerge as a potential mediator in the relationship between DT and dysfunctional eating patterns. Body uneasiness encompasses various dimensions of body dissatisfaction (i.e., dissatisfaction with physical appearance and body functionality) and general feelings of unease about body or weight (Muzi et al., 2023). In particular, two pathways could support the mediating effect of body uneasiness. On the one hand, there is considerable research linking DT to body image concerns (Waller et al., 2008). For instance, narcissism was found to be related to body checking and exercise overuse in women (Campbell and Waller, 2010). Additionally, Machiavellianism has been identified as a risk factor in the relationships between body image concerns and self-objectification (Dryden and Anderson, 2019), while positive relationships have been observed between psychopathy and wishing to be taller (Kozłowska et al., 2023). On the other hand, it is well-established that body uneasiness can drive avoidance behaviors, compulsive checking practices, detachment and/or estrangement feelings toward the body, as well as eating disorder symptoms (Zanardo et al., 2014). For instance, body uneasiness was found to be positively associated with orthorexia nervosa in females (Brytek-Matera et al., 2017) as well as a driver of different facets of eating disorders, including anorexia nervosa binge-purging type, anorexia nervosa restrictive type, and bulimia nervosa purging type (Cuzzolaro et al., 2006). Given these mechanisms, it is reasonable that people high in DT show high body uneasiness that, in turn, triggers the individual risk to engage in eating disordered patterns.

### 1.1 Aims and hypotheses

With body image and eating disorders becoming an increasing concern for today’s Western society (Mayer et al., 2024), it has become crucial to understand and identify the main factors that contribute to individual variance in dysfunctional eating. Developing a comprehensive and nuanced understanding of the role that personality traits and feelings about body image play in this field is critical for guiding and informing prevention interventions. Consequently, the current study aimed to extend the knowledge about the relationships between personality and dysfunctional eating by focusing on a less explored personality taxonomy, such as DT. In addition, this study sough to disclose the main mechanisms influencing this association, with a focus on body uneasiness. To achieve these aims, a cross-sectional study was conducted on a sample of undergraduate students under the age of 26. This age limit was selected based on the evidence suggesting a heightened risk for the onset of eating disorders during this developmental period, as well as the prevalence of narcissistic traits within this age group (Hudson et al., 2007; Twenge et al., 2008).

Participants completed a battery of measures designed to assess DT, risk of eating disorders, and different facets of body uneasiness. These include weight phobia (i.e., fear of being or becoming fat), body image concerns (i.e., worries related to physical appearance), avoidance (i.e., body image-related avoidance behaviors), compulsive self-monitoring (i.e., compulsive checking of physical appearance), and depersonalization (i.e., detachment and estrangement feelings towards one’s own body). The hypotheses were formulated as follows:

Hypothesis 1 (H1): Based on the emerging research on the relationships between DT and eating disorders (Brunett and Oberle, 2022; Hambrook and Tchanturia, 2008; Szymczak et al., 2023), this study advanced the hypothesis that all facet of DT positively correlates with the risk of eating disorders.

Hypothesis 2 (H2): Based on the logic that DT can drive different body image concerns (Campbell and Waller, 2010; Dryden and Anderson, 2019; Kozłowska et al., 2023), which, in turn, may trigger risk practices for eating (Zanardo et al., 2014), the current study hypothesized that body uneasiness mediates the relationships between all facets of DT and risk of eating disorders.

## 2 Materials and methods

### 2.1 Participants and procedure

A total of 430 individuals participated in the study. After the removal of outliers by *z*-test with ±4.0 *z*-scores as the reference values for samples larger than 100 (Mertler et al., 2021; Giancola et al., 2023b), the final sample comprised 419 participants with an average age of 21.61 years (SD = 1.95, range 18–25). Of these, 243 (58%) were female and the remaining 176 (42%) were male. In terms of education, 371 (88.5%) completed an upper secondary school, whereas 48 (11%) held a university degree. Based on the BMI formula [BMI = weight (in kg)/height^2^ (in m^2^)], 37 (8.8%) participants were underweight (<18.5 kg/m^2^), 290 (69.2%) were normal weight (18.5–24.9 kg/m^2^), 82 (19.6%) were overweight (25–29.9 kg/m^2^), and 10 (2.4%) were obese (>30 kg/m^2^). The sample was derived from a web-based cross-sectional survey on Sustainable Behaviors and Health set up by the Department of Biotechnological and Applied Clinical Sciences, University of L’Aquila. Participants were recruited from different social media (Facebook, Instagram, and WhatsApp) as well as advertisements handed out at the University of L’Aquila. Participants were provided with an online link to access a comprehensive information note about the fundamental principles of ethical scientific research and the main information regarding the nature and objectives of the study. Once participants gave their electronic consent to take part in the survey, they were requested to provide socio-demographic information. This included details such as age, gender, and years of education. Then, they were asked to fill in a set of self-report measures assessing DT, body uneasiness, and dysfunctional eating. The inclusion criteria were age from 18 to 25 years and no diagnoses of psychiatric disorders, which could show patterns of comorbidity with eating disorders, such as personality, mood, and neurotic issues (see Momen et al., 2022). Table 1 shows descriptive statistics of the study sample.

**Table 1:** Descriptive statistics of the study sample.

	Frequency (%)	*M (SD)*	*Min-Max*
Age		21.61 (1.96)	18–25
Gender			
Male	176 (42)		
Female	243 (58)		
Height (m)		1.70 (0.09)	1.50–1.93
Weight (kg)		66.72 (13.05)	41–95
BMI		22.90 (3.11)	17–31
Education (in years)		13.34 (0.96)	13–16
Income (in Euros)			
<10.000	48 (11.5)		
11.000–25.000	188 (44.9)		
26.000–50.000	113 (27.0)		
51.000–75.000	36 (8.6)		
76.000–100.000	15 (3.6)		
>100.000	19 (4.5)		

N = 419. BMI, Body mass index.

The minimum sample size was calculated using G*Power 3.1.9.7 software (Faul et al., 2007). The parameters entered were: test family: “*F* test analysis,” statistical test: “Linear multiple regression: fixed model, *R^2^* deviation from zero,” type of analysis: “*A priori*: Compute required sample size – given *α*, power and effect size,” *α* err prob. = 0.05, power (1-*β* err prob) = 0.95, mean effect size *f2* = 0.15 (medium effect), and a maximum number of predictors = 9 (one independent variable, five mediators, and three covariates, such as age, gender, and education). G*Power recommended a minimum sample size of 166. The study was elaborated in accordance with the Declaration of Helsinki and was subjected to the review and approval of the Ethics Committee of the University of L’Aquila (protocol number: 119943). Survey participants involved in this research were not rewarded; all participants provided electronic informed consent, and anonymity was guaranteed.

### 2.2 Measures

#### 2.2.1 Dark triad

The Dark Triad Dirty Dozen (DTDD; Schimmenti et al., 2019) is a brief self-assessment tool comprising 12 items rated on a 5-point Likert scale, with responses ranging from 0 (not at all) to 4 (very much). The DTDD allows for evaluating Machiavellianism, psychoticism, and narcissism. Like previous studies, the total score of the DT was calculated by taking the average of the standardized values for each trait (e.g., Giancola et al., 2024a). In the current study, Cronbach’s alpha for the DTDD total score was 0.91.

#### 2.2.2 Body uneasiness

The Body Uneasiness Test A (BUT-A; Cuzzolaro et al., 2006) is a multidimensional self-report measure assessing body uneasiness. The BUT-A consists of 34 items that measure Weight Phobia (WP), Body Image Concerns (BIC), Avoidance (A), Compulsive Self-Monitoring (CSM), and Depersonalization (D). Each item is rated on a 6-point Likert scale from 0 (never) to 5 (always). This measure also yields a total test score known as the Global Severity Index (GSI). We considered the WP, BIC, A, CSM, and D subscales to understand body image dissatisfaction better. Higher scores indicate more body uneasiness. In previous research, the BUT-A showed good psychometric properties (Carpinelli et al., 2023). In this sample, Cronbach’s alpha ranged from 0.81 to 0.92.

#### 2.2.3 Risk of eating disorders

The Eating Attitudes Test-26 (EAT-26; Dotti and Lazzari, 1998; Garner et al., 1982) is a 26-question self-assessment to identify the risk of eating disorders. The EAT-26 consists of a 6-point Likert-type scale, ranging from 0 (never) to 3 (always). Items 1–25 were scored as follows: “always” = 3, “usually” = 2, “often” = 1, and “sometimes/rarely/never” = 0. Conversely, item 26 was scored in a reversed manner, wherein “always/usually often” = 0, “sometimes” = 1, “rarely” = 2, and “never” = 3. The total score of the EAT-26 ranges from 0 to 78, with higher scores reflecting a higher risk of eating disorders. A threshold of 20 or higher is widely used to detect the presence of clinically significant pathology associated with eating disorders (Garfinkel and Newman, 2001). The EAT-26 showed excellent psychometric properties in previous research (Calugi et al., 2018), and in the present study, Cronbach’s alpha was 0.90.

#### 2.2.4 Socio-demographic variables

Participants provided socio-demographic information, including age, gender (dummy coded where 0 = female and 1 = male), education (in years), and annual income.

#### 2.2.5 Body mass index

Self-reported height and weight were used to compute participants’ body mass index (BMI).

### 2.3 Statistical analysis

Statistical analysis was performed using SPSS Statistics version 26 for Windows (IBM Corporation, Armonk, New York, USA, 2019) and MACRO Process version 4.0 (Hayes, 2022). Descriptive statistics, common method bias (CMB) check, and bivariate correlation analyses were run to evaluate data preliminarily, whereas mediation analysis was performed, setting Model 4 in the MACRO Process. The significance of the mediating effects was assessed using 5,000 resamples of bootstrapped estimates with 95% bias-corrected confidence intervals – CIs, which must not cross zero to satisfy the criteria of mediation (Preacher and Hayes, 2008; Giancola et al., 2023c; Giancola et al., 2023d). All significance was set to *p* < 0.05.

## 3 Results

Statistical analysis revealed that all variables were not normally distributed. In addition, Harman’s single-factor test (Harman, 1967) suggested that the variance explained by a single-factor exploratory model was 38.13%. Therefore, no CMB problems were detected (test critical threshold ≥50%). A single-factor CFA was performed to test the CMB further (Podsakoff et al., 2003). The results indicated that the fit index was not good (χ2/df = 16.10, CFI = 0.69, TLI = 0.63, RMSEA = 0.19), suggesting no CMB problems. Table 2 reports correlations among all study variables.

**Table 2:** Means, standard deviations, and inter-correlations among study variables.

	1.	2.	3.	4.	5.	6.	7.	8.	9.	10.	11.	12.	13.
Age	1												
Gender	0.14**	1											
Education	0.41**	−0.06	1										
BMI	0.02	0.47**	−0.11	1									
Machiavellianism	−0.04	0.16**	0.04	−0.10*	1								
Psychopathy	0.07	0.13**	0.04	−0.07	0.60**	1							
Narcissism	0.03	0.16**	0.01	−0.04	0.36**	0.39**	1						
Weight phobia	0.02	−0.33**	0.11*	−0.25**	0.07	0.03	0.21**	1					
Body image concerns	−0.06	−0.39**	0.05	−0.36**	0.15**	0.16**	0.18**	0.81**	1				
Avoidance	0.05	−0.31**	0.07	−0.21**	0.03	−0.03	0.21**	0.75**	0.65**	1			
Compulsive self-monitoring	−0.02	−0.24**	0.02	−0.28**	0.35**	0.25**	0.37**	0.59**	0.71**	0.49**	1		
Depersonalization	0.03	−0.32**	0.06	−0.27**	0.09	0.07	0.21**	0.73**	0.72**	0.77**	0.65**	1	
Risk of eating disorders	0.01	−0.08	0.08	−0.38**	0.04	0.09	0.17**	0.34**	0.48**	0.34**	0.33**	0.39**	1

*N* = 419, gender is dummy coded (0 = F; 1 = M), BMI, Body Mass Index. *p < 0.05 (two tailed); **p < 0.01 (two tailed).

Mediation analyses showed a significant direct effect of Machiavellianism on the risk of eating disorders (*B* = −0.87, *SE* = 0.40, *t* = −2.17, 95% *CI* [−1.6533, −0.0806]), while no mediating effects of weight phobia, body image concerns, avoidance, compulsive self-monitoring, and depersonalization were found (see Table 3, Model A). In addition, results indicated that the direct effect of psychopathy on risk of eating disorders was not significant (*B* = 0.57, *SE* = 0.33, *t* = 1.73, 95% *CI* [−0.0760, 1.2064]) with no mediating effects of weight phobia, body image concerns, avoidance, compulsive self-monitoring, and depersonalization (see Table 3, Model B). Finally, results revealed that the direct effect of narcissism on the risk of eating disorders was not significant (*B* = 0.76, *SE* = 0.39, *t* = 1.94, 95% *CI* [−0.0085, 1.5344]). However, the analysis showed that among the different facets of body uneasiness, only body image concerns mediated the interplay between narcissism and risk of eating disorders (see Table 3, Model C).

**Table 3:** The indirect effects of Machiavellianism, psychopathy, and narcissism on dysfunctional eating.

Path	*B*	SE	BootLLCI	BootULCI
Model A				
Machiavellianism → Weight phobia → Risk of eating disorders	0.00	0.06	−0.1270	0.1320
Machiavellianism → Body image concerns → Risk of eating disorders	0.18	0.22	−0.2315	0.6381
Machiavellianism → Avoidance → Risk of eating disorders	−0.01	0.06	−0.1628	0.0864
Machiavellianism → Compulsive self-monitoring → Risk of eating disorders	0.04	0.14	−0.2451	0.3238
Machiavellianism → Depersonalization → Risk of eating disorders	−0.01	0.05	−0.1019	0.1073
Model B				
Psychopathy → Weight phobia → Risk of eating disorders	0.01	0.05	−0.1075	0.1314
Psychopathy → Body image concerns → Risk of eating disorders	0.26	0.18	−0.0522	0.6512
Psychopathy → Avoidance → Risk of eating disorders	−0.03	0.09	−0.2377	0.1365
Psychopathy → Compulsive self-monitoring → Risk of eating disorders	0.00	0.04	−0.0743	0.0828
Psychopathy → Depersonalization → Risk of eating disorders	−0.01	0.05	−0.1326	0.1042
Model C				
Narcissism → Weight phobia → Risk of eating disorders	0.01	0.25	−0.5067	0.4816
Narcissism → Body image concerns → Risk of eating disorders	0.51	0.24	0.1225	1.0406
Narcissism → Avoidance → Risk of eating disorders	0.12	0.32	−0.4262	0.8355
Narcissism → Compulsive self-monitoring → Risk of eating disorders	0.08	0.26	−0.4346	0.5961
Narcissism → Depersonalization → Risk of eating disorders	0.08	0.27	−0.4863	0.6080

*N* = 419; For the Model A covariates are age, gender, education, BMI, psychopathy, and narcissism. For Model B covariates are age, gender, education, BMI, Machiavellianism, and narcissism. For Model C covariates are age gender, education, BMI, Machiavellianism, and psychopathy. The explain variance of each model is as follows: Model A: *R*^2^ = 0.44 [*F*(12,406) = 26.24, *p* < 0.001; Model B: *R*^2^ = 0.44 *F*(12,406) = 26.24, *p* < 0.001; Model C: *R*^2^ = 0.44 *F*(12,406) = 26.24, *p* < 0.001].

## 4 Discussion

The purpose of this study was to extend the knowledge about the interplay between personality traits and dysfunctional eating habits with a focus on DT and the risk of eating disorders. Furthermore, this study delved into the main mechanisms that could impact this association, addressing the potential mediating effect of body uneasiness. This latter was evaluated in terms of weight phobia, body image concerns, avoidance, compulsive self-monitoring, and depersonalization.

Results showed that only narcissism was positively associated with the risk of eating disorders, partially confirming H1 (i.e., DT positively correlates with the risk of eating disorders). These findings substantiated the view that among the three facets of DT, narcissism consistently explains individual differences in engaging risk eating practices. Indeed, unlike individuals with high Machiavellianism and psychopathy, people who exhibit high levels of narcissism are more likely to prioritize appearance (Fox and Rooney, 2015). This narcissistic tendency aligns with the view that individuals with high narcissism tend to objectify their bodies, viewing themselves as an object that needs to be admired and desired by others (Carrotte and Anderson, 2019). Given this mechanism, these individuals engage in appearance-focused behaviours, such as altering their photographs on social media, overusing exercise, grooming, and cosmetic procedures, as well as adopting extreme dieting to maintain and bolster their desired and inflated self-image grandiosity, superiority, and status, as well as strengthen attractiveness and admiration from others (Holtzman and Strube, 2010).

In addition, mediation analysis revealed that among the five dimensions of body uneasiness (i.e., weight phobia, body image concerns, avoidance, compulsive self-monitoring, and depersonalization), only weight phobia mediated the association between narcissism and risk of eating disorders. These findings partially confirmed H2 (i.e., body uneasiness mediates the relationships between DT and the risk of eating disorders). Several explanations could support the evidence on the mediating effect of body image concerns in the association between narcissism and the risk of eating disorders. First, people who exhibit high levels of narcissism usually show not only an inflated self-view, sense of self-importance, and superiority but also unrealistic expectations for themselves (Campbell and Foster, 2011). These feelings entail a broad range of domains, including physical appearance, which could lead these individuals to develop an ideal physical appearance (Carrotte and Anderson, 2019). Moreover, individuals with high levels of narcissism are particularly susceptible to the influence of societal beauty norms and the pressure to conform to unrealistic standards of physical perfection (Cowan-Jenssen and Goodison, 2009). They often believe adhering to these standards can showcase their superiority and value to others (Flett et al., 2014). Nevertheless, when the ideal image does not align with the actual body image, individuals with high levels of narcissism may experience significant dissatisfaction with their bodies, which can contribute to the development of unhealthy eating habits. These practices may represent a desperate attempt to reach their unachievable body ideal and, consequently, a means to foster their superiority and status.

Second, as previously mentioned, people with high narcissism often show high levels of self-objectification. This tendency can lead people high in narcissism to a distorted perception of the body and a dysfunctional preoccupation with physical appearance that could reinforce their self-objectifying perspective, nurturing the desire to manipulate the body in order to gain admiration and attention from others. Accordingly, dysfunctional eating attitudes may emerge as a means of exerting control over and enhancing the perceived attractiveness of the objectified self. This mechanism could be particularly grounded in individuals who exhibit high narcissism due to their tendency to be affected by societal and cultural ideal standards of beauty (Bourdier et al., 2018). Third, previous research suggested that people with high levels of narcissism may have difficulty regulating and managing negative emotions, feelings, and beliefs (Mojsa-Kaja et al., 2021). Body image concerns can exacerbate these difficulties, contributing to feelings of inadequacy, shame, and anxiety about appearance. In order to mitigate these negative feelings as well as maintain a sense of authority and control over their appearance, individuals who exhibit high narcissism may engage in dysfunctional eating practices as a maladaptive coping mechanism.

The present research show a few limitations worth mentioning. First, this study adopted a cross-sectional online survey design, which addresses the associations among the study variables at a single time. As this design does not allow for establishing cause and effect inferences, future research would provide more evidence about the directionality of the relationships between DT, body uneasiness, and the risk of eating disorders through a longitudinal survey design. In addition, this study used an online survey accessible to the public, allowing anyone to complete the survey voluntarily by clicking a link. While online surveys have been widely used in previous health-related research (Giancola et al., 2024c), they are vulnerable to several types of bias, including self-selection bias (Bethlehem, 2010). This latter occurs when individuals determine whether to participate in research based on their own preferences or characteristics, rising to the possibility that the sample may not adequately represent the population (Stone et al., 2024). As the self-selection bias can undermine the generalizability of the results, future research should confirm the findings provided by this study through probability sampling instead of a self-selected sample. Second, this study mainly focused on the DT, overlooking some facets of the dark side of personality, including factor 2 psychopathy, vulnerable narcissism, and borderline symptomatology, otherwise known as the vulnerable DT (Maheux-Caron et al., 2024; Miller et al., 2010). This implies that the findings provided by the present study may under-represent the full spectrum of dark personality dynamics associated with the risk of eating disorders. For instance, interpersonal hypersensitivity and feelings of shame of vulnerable narcissism, as well as interpersonal hypersensitivity and feelings of shame of factor 2 psychopathy and self-destruction, impulsivity, and affective instability of borderline symptomatology could play a crucial role in the psychological processes associated with the risk of eating disorders. Given that, a more granular approach to dark personalities when addressing the risk of eating disorders is envisaged. Finally, due to the heightened risk for the onset of eating disorders and the prevalence of narcissistic traits within 18–25 years old (Hudson et al., 2007; Twenge et al., 2008), this study focused on a sample of youth. However, broadening the demographic scope to older adults or people with different educational backgrounds might reveal more distinct patterns the risk of eating disorders.

Despite these limitations, the current study offers some theoretical and practical implications that warrant consideration. From a theoretical standpoint, it provides further evidence on the critical role of personality in explaining individual differences in the risk of eating disorders, by focusing on an under-explored personality taxonomy, such as DT. Specifically, this study reveals that among the three facets of DT, only narcissism showed a positive association with the risk of eating disorders, suggesting the importance of focusing on this personality trait when evaluating the risk of eating disorders. In terms of clinical practice, the findings carry notable implications for assessing and treating eating disorders with positive consequences for individual well-being and positive youth development (Giancola et al., 2021). In particular, to provide a more tailored intervention, clinicians should pay specific attention to the presence of narcissistic tendencies, particularly when such patterns coincide with body image concerns. In addition, clinicians could develop psychoeducational modules that may help individuals with narcissistic tendencies and body image concerns build a more realistic and less body-focused sense of self-worth. Cognitive-behavioral approaches could help challenge the distorted beliefs about appearance and self-image, while psychodynamic or interpersonal therapies might help to explore the underlying insecurities that fuel narcissistic grandiosity. In addition to addressing narcissistic tendencies and body image concerns, clinicians should also consider the potential role of anger and hostility in treatment dropout and non-compliance. Indeed, previous research revealed that anger and hostility, which are commonly associated with DT, can significantly contribute to dropout rates in therapeutic interventions and follow-up studies (Todisco et al., 2023). These emotions may cause individuals to disengage from treatment, particularly in interventions that challenge their self-perceptions or demand emotional vulnerability. Therefore, clinicians should identify anger and hostility early in the therapeutic process and use targeted strategies to manage them. This should improve treatment outcomes and ultimately foster a more sustained engagement in the intervention.

## Data Availability

The raw data supporting the conclusions of this article will be made available by the authors, without undue reservation.
